# Correlation between dysbiosis of vaginal microecology and endometriosis: A systematic review and meta-analysis

**DOI:** 10.1371/journal.pone.0306780

**Published:** 2024-07-08

**Authors:** Xuemei Qing, Min Xie, Ping Liu, Ou Feng, Hong Leng, Hongying Guo, Yong Zhang, Ying Ma, Wenxin Zheng

**Affiliations:** 1 Department of Obstetrics and Gynecology, Qingbaijiang District People’s Hospital, Chengdu, Sichuan, China; 2 Department of Obstetrics and Gynecology, Southwest Medical University, Luzhou, Sichuan, China; 3 Department of Obstetrics and Gynecology, West China Second University Hospital, Sichuan University, Chengdu, Sichuan, China; 4 Department of Obstetrics and Gynecology, Mianyang Central Hospital, Mianyang, Sichuan, China; 5 Department of Obstetrics and Gynecology, Chengdu Medical College, Chengdu, Sichuan, China; 6 Department of Obstetrics and Gynecology, Department of Pathology, Harold C Simmons Comprehensive Cancer Center at the University of Texas Southwestern Medical Center, Dallas, TX, United States of America; University of Bremen: Universitat Bremen, GERMANY

## Abstract

**Background:**

Endometriosis, a complex gynecological condition, involves inflammation and immune dysregulation. The vaginal microbiota, characterized by its diversity, is an integral part of the vaginal microecology—interacting with vaginal anatomy, the endocrine system, and local mucosal immunity. Imbalances in this microecology are known to precipitate various inflammatory diseases. Despite extensive research, the connection between vaginal microbiota dysbiosis and endometriosis remains a subject of debate. Our study assesses the association between vaginal microecology dysbiosis and endometriosis.

**Methods:**

We systematically searched major electronic databases in English, including Embase, PubMed, The Cochrane Library, MEDLINE (Ovid), BIOSIS (Ovid), China National Knowledge Infrastructure (CNKI), and Wanfang, up to August 15, 2023. Selected articles underwent screening based on predefined inclusion and exclusion criteria. Normal vaginal microecology was defined as a negative Amsel/Spiegel test or Nugent score of 0–3, or *Lactobacillus* predominance determined by 16S rRNA gene amplification sequencing. Deviations from this norm were classified as dysbiosis, further categorized into bacterial vaginosis (BV) and intermediate BV. Data analysis utilized Revman 5.4, with effect sizes presented as Odds Ratios (OR) and 95% Confidence Intervals (CI).

**Results:**

Out of 1081 articles, eight met the inclusion criteria. Utilizing fixed-effect models due to low heterogeneity, the analysis revealed a positive association between dysbiosis and endometriosis (OR = 1.17, 95% CI 0.81–1.70; I^2^ = 0%), but showed a slight negative association between normal vaginal microecology with endometriosis (OR = 0.90, 95% CI 0.55–1.46; I^2^ = 29%). However, the association was not significant. Subgroup and sensitivity analyses corroborated the stability of these associations.

**Conclusion:**

A positive correlation exists between vaginal microecology dysbiosis and endometriosis, notably with intermediate BV. However, the mechanisms underpinning this relationship remain elusive, highlighting the need for further research to overcome limitations.

**Trial registration:**

Registration number: CRD42023445163.

## Introduction

Endometriosis, defined by the presence of endometrial-like tissue outside the uterus, is a chronic estrogen-dependent condition marked by its inflammatory nature. Affecting approximately 10% of women in their reproductive years, endometriosis can severely impact the quality of life with symptoms ranging from severe dysmenorrhea to chronic pelvic pain, though some individuals remain asymptomatic, due to its highly heterogeneous [1]. The etiology of endometriosis is multifactorial, with theories encompassing retrograde menstruation, hormonal imbalances, immune alterations, genetic and epigenetic factors, and even stem cell irregularities—each potentially playing a role in its onset and progression [2].

The pathogenesis of endometriosis is intricately linked with immunological changes; however, the specifics of this relationship are yet to be fully understood. Studies in animal models have demonstrated that endometriosis can drive inflammation via increased production of inflammatory mediators, potentially due to shifts towards inflammatory immune and mucosal microbial profiles [3]. The “bacterial contamination hypothesis” suggests a role for bacterial endotoxins in the pathogenesis of endometriosis, with studies showing significant *Escherichia coli* contamination in menstrual blood and peritoneal fluid of patients with endometriosis [4]. Notably, the presence of *Fusobacterium* is markedly higher in the endometrium of endometriosis patients, suggesting bacterial infection could be a contributing factor [5]. A national cohort study showed that lower genital tract infections can be an independent risk factor for endometriosis [6].

Female vaginal microecology is an ecosystem composed of vaginal microbiota (VMB), host endocrine system, vaginal anatomy, and local immune system in a dynamic balance. VMB refers to the microorganisms that are commonly found in the vagina. Microbial populations isolated from the vagina include *Lactobacillus*, *Gardnerella vaginalis*, *Prevotella bivia*, *Atopobium spp*., *Mobiluncus*, *Bacteroidetes*, *Bifidobacterium spp*., *Escherichia coli*, *Candida albicans*, *Trichomonas vaginalis*, *Actinobacillus spp*., and *Sheathed Anaerobic Coccobacillus*, as well as other rare bacterial and non-bacterial pathogens. The VMB is resistant to pathogens associated with infectious diseases of the genitourinary tract and sexually transmitted diseases. The vaginal microbiota is an important barrier protecting the host from a variety of bacterial, fungal, viral, and other infections [7]. When the normal vaginal flora is disrupted and the micro-ecological environment is altered, dysbiosis is likely to occur and even lead to a variety of vaginal infectious diseases.

Conversely, bacterial vaginosis (BV), the most prevalent vaginal dysbiosis, is characterized by a decrease in *Lactobacillus* and an increase in anaerobic bacteria [8]. Diagnostic methods for BV have evolved from direct Gram staining to the Nugent score, deemed the “laboratory gold standard” [9]. Advances in molecular techniques have shed light on the diverse pathogens associated with BV, with *Gardnerella vaginalis* and *Prevotella bivia* identified as primary colonizers, and various other anaerobes as secondary ones [10–13]. Importantly, *Gardnerella vaginalis* and other anaerobes initially adhere to the vaginal wall and then form a biofilm that establishes a symbiotic and synergistic relationship. The biofilm formed is closely related to the onset and recurrence of BV and inflammation induced by the pathogenic bacteria that elicit innate and adaptive immune responses and evasion of the host immune system, which can resist standard therapies [14–16]. High-throughput sequencing has further refined our understanding, categorizing the vaginal microbiota into five community state types based on the predominant *Lactobacillus* species [17, 18]. The beneficial activity of *Lactobacillus* is not caused by a single Lactobacillus species but by its multi-microbial interactions. *L*. *jensenii*, *L*. *gasseri*, *L*. *iners*, and *L*. *acidophilus* were shown to be a potent multi-microbial consortium. However, the probiotic activity the multi-microbial consortium promotes remains unknown [19]. These microbiotas play a crucial defensive role, even minor imbalances can lead to disease [20].

Studies correlate BV with an increased susceptibility to inflammatory disorders, infertility, and lower genital tract infections, which are also risk factors for endometriosis [21–25]. Recent evidence suggests that disorders of the vaginal microbiota and inflammatory processes may influence the development of both pelvic inflammatory disease (PID) and endometriosis, with endometriosis patients showing a higher prevalence of recurrent vaginitis and Vulvovaginal Candidiasis [26, 27]. A pilot study highlighted an increased presence of *Atopobium spp*. in the lower genital tract of Chinese endometriosis patients with adenomyosis [28]. While a recent systematic review has explored the link between the microbiome and endometriosis, it included animal studies and research on intestinal microbiota [29]. Therefore, our study narrows its focus exclusively to human studies concerning vaginal microbiota to assess its relationship with endometriosis.

## Methods

We executed a systematic review and meta-analysis in strict compliance with the Preferred Reporting Items for Systematic Reviews and Meta-Analyses (PRISMA) guidelines, ensuring transparency and reproducibility of our research methodology [30]. The protocol was proactively registered with the International Prospective Register of Systematic Reviews (PROSPERO) in July 2023 to guarantee the integrity of our review process, with the assigned registration number CRD42023445163 (PROSPERO (york.ac.uk)).

### Search strategy

The search strategy was collaboratively formulated and executed by authors Qing and Xie. We conducted a comprehensive search of the electronic databases Embase, PubMed, The Cochrane Library, MEDLINE (Ovid), BIOSIS (Ovid), China National Knowledge Infrastructure (CNKI), and Wanfang database. The search spanned from each database’s inception up to the cutoff date of August 15, 2023, and was restricted to English-language publications.

Our search terms were carefully selected to encapsulate the relationship between endometriosis and vaginal microecology. We used a combination of keywords and MeSH terms: “Endometriosis” in conjunction with “Lower Genital Tract” or “Vagina”, and “Dysbiosis” or “Inflammation”, “Infections”, “Bacterial Vaginosis”, “Aerobic Vaginitis”, “Vulvovaginal Candidiasis”, or “Trichomonas Vaginitis”. Each term was used alongside its respective synonyms to ensure a broad and thorough retrieval of relevant literature.

In addition to electronic database searches, we conducted a manual search through the reference lists of all identified articles to uncover further pertinent studies. This dual-faceted approach aimed to yield an exhaustive collection of sources pertinent to our research question. The detailed search strategy, including the specific combinations and permutations of search terms used, has been documented in S1 File.

### Literature selection

To maintain a high standard of scientific rigor, we established stringent criteria for the inclusion and exclusion of studies. Our aim was to ensure that only the most relevant and reliable data were considered for this review.

### Inclusion criteria

1. **Study population:** Research must compare individuals diagnosed with endometriosis to a control group without the condition, with a focus on their vaginal microbiota.
2. **Study design:** Only human observational studies providing original data were considered.
3. **Diagnostic assessment:** Studies must employ vaginal microbiota assays using 16S rRNA gene amplification sequencing, or must assess vaginal microecology using the Nugent score or Amsel/Spiegel criteria.
4. **Data availability:** The study must present extractable data on vaginal microbiota.

### Exclusion criteria

1. **Publication type:** We excluded duplicate publications, reviews, meta-analyses, conference abstracts, letters to the editor, guidelines, consensus statements, case reports, and case series.
2. **Study design:** Animal studies, in vitro experiments, intervention trials, and studies without a control group were not considered.
3. **Language:** Non-English language studies were excluded to ensure the interpretability and verifiability of data.

Studies meeting the inclusion criteria underwent a full-text review to confirm their eligibility. This process was carried out by two independent reviewers, with any disagreements resolved through consensus or by a third-party adjudication. This approach was designed to minimize selection bias and to ensure that only the most methodologically sound studies were included in our analysis.

### Literature selection

The literature screening process was meticulously executed by two reviewers, Qing and Xie, who independently evaluated the titles and abstracts of retrieved articles for relevance. This initial phase was instrumental in identifying publications that potentially met our research objectives. Subsequently, these selected articles underwent a rigorous review based on the established inclusion and exclusion criteria. The process was designed to ensure a methodical and unbiased selection of studies for further analysis. In instances of divergent opinions between the two primary reviewers, a consultative discussion with a third author, Ma, was the deciding factor in resolving any discrepancies.

### Quality assessment

The methodological quality of each included study was carefully appraised independently by Qing and Xie. For cohort and case-control studies, the Newcastle-Ottawa Scale (NOS) was employed as the evaluation tool [31], while the Agency for Healthcare Research and Quality (AHRQ) checklist was utilized for cross-sectional studies [32] This dual-tool approach allowed for a comprehensive quality assessment across different study designs. Disagreements in quality scoring were addressed through a consensus-seeking discussion or, if necessary, by deferring the final judgment to Ma. The specifics of these quality assessments, including the scoring criteria and outcomes, have been thoroughly documented in S2 File.

### Quality assessment instruments

For the critical appraisal of the included studies, two established instruments were utilized.

*Newcastle-Ottawa Scale (NOS)*. This scale evaluates three core aspects: selection of the study groups, group comparability, and the determination of either the exposure or outcome of interest for case-control or cohort studies respectively. Employing a semi-quantitative star system, the NOS allocates a maximum of nine stars across eight detailed criteria within these categories.

*Agency for Healthcare Research and Quality (AHRQ) checklist*. This checklist comprises eleven items that scrutinize various dimensions of study quality, including clarity in information sourcing, explicitness in patient selection, and management of study biases. Responses to each item are graded as "Yes", "No", or "Unclear", corresponding to scores of 1, 0, or 0, respectively. The aggregate score classifies the studies into low (0–3), medium (4–7), or high (8–11) quality categories.

### Data extraction process

Data extraction was meticulously conducted by Qing and Xie, extracting crucial details such as the first author’s name, publication date, study locale, methodology, demographics, sample sizes, and outcomes related to the prevalence of *Lactobacillus*, BVAB/CST IV, and BV. Disagreements were amicably resolved through discussion or consultation with a third author, Ma. Definitions for normal vaginal microecology and dysbiosis were grounded in Amsel/Spiegel test outcomes, Nugent scores, and 16S rRNA gene amplification sequencing results. An extended table detailing the characteristics of the included studies (S1 Table) provides further insights.

### Statistical analysis framework

Our statistical analysis aimed to elucidate the relationship between vaginal microecology dysbiosis and endometriosis. The Mantel-Haenszel method was employed to compute pooled Odds Ratios (ORs) and 95% Confidence Intervals (CIs). Heterogeneity was assessed using Cochrane’s Q test and the I^2^ statistic, guiding the choice between fixed or random-effects models based on the I^2^ value (fixed model for I^2^ <50%). We further dissected the data through subgroup analyses based on study design, geographic region, control group characteristics, diagnostic methods, and degrees of dysbiosis. Results from these analyses are available in the S3 File.

To verify the robustness of our findings, sensitivity analyses were performed by systematically omitting each study. Publication bias was evaluated through funnel plot inspections. All statistical computations were facilitated by Revman 5.4, with a two-tailed P-value threshold of <0.05 set for statistical significance.

## Results

### Study selection overview

Our comprehensive search yielded 1081 English language articles deemed initially eligible for inclusion. Utilizing Endnote’s intelligent screening capabilities, we systematically excluded non-relevant literature: 158 duplicates, 237 reviews and meta-analyses, 79 conference proceedings, 6 replies or letters, 7 guidelines, 66 case reports, 34 animal studies, and 15 intervention trials. Subsequent screening of titles and abstracts resulted in the removal of an additional 462 articles that did not meet our research criteria.

A more detailed evaluation of the remaining 17 articles was conducted through full-text reviews. This phase led to further exclusions: 1 article only had an abstract; 1 article was a methodological protocol without results; 1 abstract had been previously published in the same study; 2 articles examined the Human Papillomavirus in endometriosis patients; 1 article investigated the prevalence of endometriosis in lower genital tract infections; 1 was a preliminary pilot study; 1 lacked a control group. The last excluded article reported the prevalence of Vulvovaginal Candidiasis in patients with endometriosis, however, it couldn’t be analyzed itself just for one. Surprisingly, no literature matched the incidence of Aerobic Vaginitis or Trichomonas Vaginitis with endometriosis.

**Fig 1:**
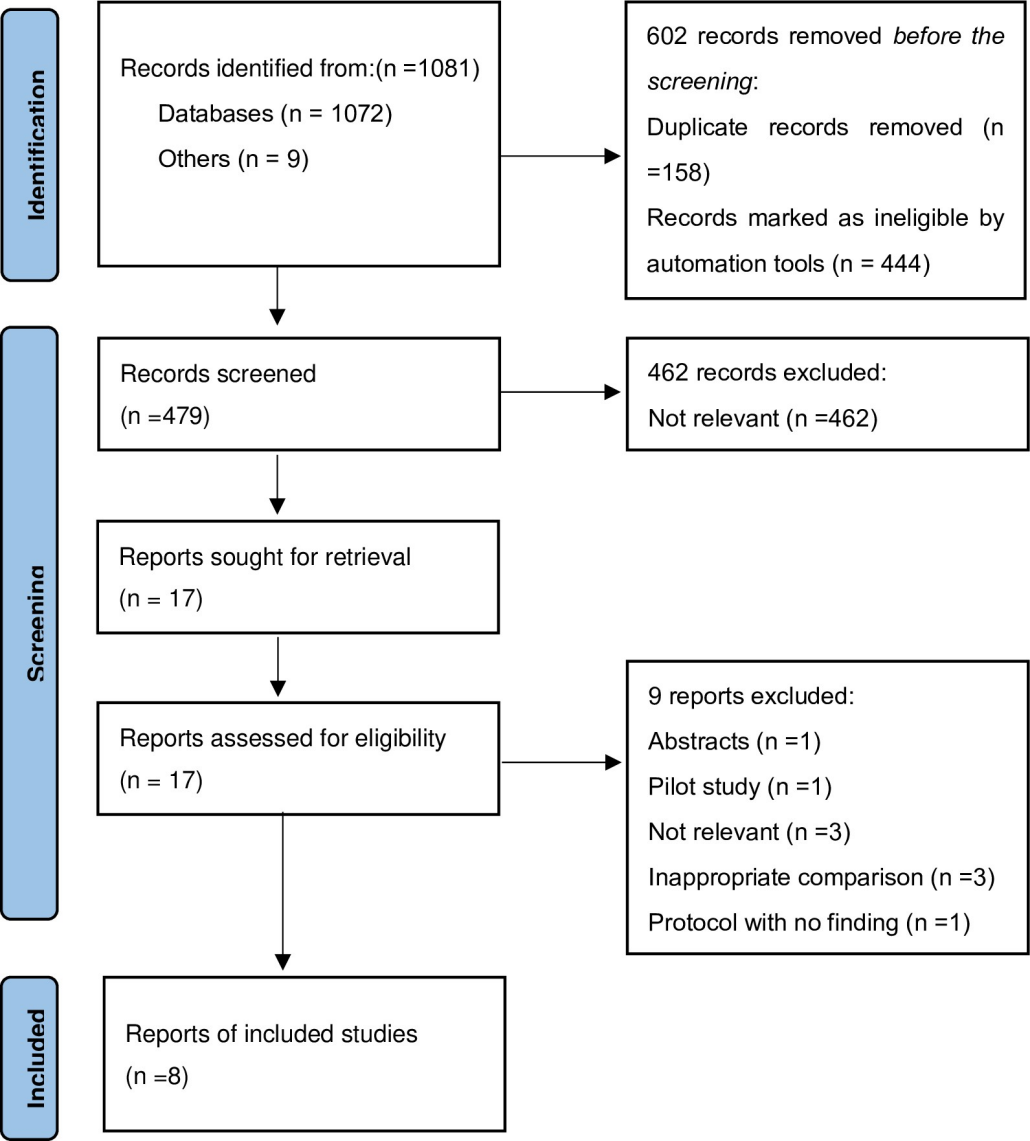
PRISMA flow diagram of study selection.

After this rigorous process, we ultimately included eight studies that met all our criteria. The progression of our study selection is visually represented in a flow diagram (Fig 1), providing a clear and concise overview of the literature filtration process.

### Basic characteristics of included studies

The eight studies that met our inclusion criteria represent a diverse collection of research designs: two cohort studies [33, 34], three case-control studies [35–37], and three cross-sectional studies [38–40]. Geographically, these studies span several countries, with two originating from China [37, 40], and one each from the United States [39], the United Kingdom [38], Australia [35], Japan [36], Egypt [33], and Turkey [34].

In total, these studies encompassed 1063 participants, divided between 300 individuals with endometriosis in the endometriosis group and 763 in the control group. The temporal alignment of data collection for endometriosis and vaginal microbiota poses challenges in establishing a clear causal link between these variables. Over half of the studies did not account for the stage of endometriosis, with only three reports providing details on the disease stage [34, 37, 39]. The cohorts also included a study focused on endometriosis concurrent with Chronic Pelvic Pain Syndrome [40], and two studies that explored the association of endometriosis -infertility with BV [33, 38].

Variability was also observed in the composition of the control groups, with some studies including asymptomatic women, fertile women, and healthy women [33–35, 38–40], while others compared against benign gynecologic conditions like uterine fibroids and ovarian cysts [36, 37]. As for the microbiota detection methods, most studies utilized 16S rRNA gene sequencing [34–37, 39, 40], with two relying on modified Spiegel’s criteria [33, 38]. The sample sites were predominantly vaginal, with four studies specifically sampling from the posterior fornix [33–35, 37–40], and one study using cervical mucus [36]. The types of Bacterial Vaginosis-Associated Bacteria (BVAB) identified varied slightly among studies, with one identifying both *Gardnerella vaginalis* (GV) and *Prevotella bivia* [30], two identifying GV alone [34, 37], and three reporting Community State Type IV (CST IV) [35, 39, 40]. The comprehensive details of these studies are presented in Table 1 and the summaries in Table 2.

**Table 1:** Basic characteristics of included studies.

Study	Country	Study design	Sample size (E/C)	Diagnosis methods	sample type	*Lactobacillus*	BVAB / CST IV	BV
Kanoko Akiyama 2019 [36]	Japan	case-control	30/39	16S rRNA gene sequencing	cervical mucus	12/20	2/4	/
Baris Ata 2019 [34]	Turkey	cohort	14/14	16S rRNA gene sequencing	vaginal swabs	12/11	2/ 1	/
Xiaopei Chao 2021 [40]	China	cross-sectional	37/66	16S rRNA gene sequencing	posterior fornix	20/47	16/19	/
Allison R. Perrotta 2020 [39]	USA and Brazil	cross-sectional	27/18	16S rRNA gene sequencing	vaginal samples	24/14	3/4	/
Rasheed M. Salah 2013 [33]	Egypt	cohort	88/382	modified Spiegel’s criteria	posterior fornix	/	/	17/59
Bryan A. Wee 2017 [35]	Australia	Case-control	12/19	16S rRNA gene sequencing	posterior fornix	11/13	4/6	/
Weixia Wei 2020 [37]	China	case-control	36/14	16S rRNA gene sequencing	posterior fornix	26/9	3/0	/
Janet D. Wilson 2002 [38]	UK	cross-sectional	56/211	Modified Spiegel’s criteria	vaginal samples	/	/	7/33

Note: **E**: Endometriosis group; **C**: Control group; **BV:** Bacterial vaginosis**; BVAB**: Bacterial vaginosis-associated bacteria; **CST**: Community status type.

**Table 2:** The summaries of included studies.

Study	Report form	Study group	Control group	Time of sample collection	Main findings
Kanoko Akiyama 2019	laparoscopic surgery	endometriosis	fibroids or benign ovarian tumor	before laparoscopic surgery	In addition to predominant *Lactobacilli spp*., the populations of *Corynebacterium*, *Enterobacteriaceae*, *Flavobacterium*, *Pseudomonas*, and *Streptococcus* were increased in the endometriosis group. *Enterobacteriaceae* and *Streptococcus* were identified as the more significant candidates in the endometriosis group by real-time PCR. NGS analysis showed that the distribution of microbiota was similar in the cervical mucus of women with and without endometriosis regardless of the menstrual phase. The prevalence rate of *Prevotella* and *Gardnerella* displayed no difference.
Baris Ata 2019	histologic diagnosis	endometriosis	asymptomatic reproductive-aged women	on the evening before surgery	In endometriosis patients, 84.6% were *Lactobacillus* and 10.5% *Gardnerella*, making 95.1% of the total bacteria. In the control group, *Lactobacillus* comprised 80.2% and *Gardnerella* 7.3% of total bacteria. For vaginal samples, at the genus level, the complete absence of *Gardnerella* and *Atopobium spp*. in the endometriosis group was noteworthy.
Xiaopei Chao 2021	exploratory laparoscopy or pathology	endometriosis/ AM with CPP	without endometriosis/ AM and CPP	on the first visit to the clinics	At the species level, endometriosis/AM-associated CPP was found to be associated with a predominance of *Clostridium butyricum*, *Clostridium disporicum*, *Alloscardovia omnicolens*, and *Veillonella montpellierensis*, and concomitant paucity of *Lactobacillus jensenii*, *Lactobacillus reuteri*, and *Lactobacillus iners*. The vaginal microbiome of patients with endometriosis /AM-associated CPP shows significantly higher alpha diversity, as well as higher counts of *Clostridium butyricum*, *Clostridium disporicum*, *Alloscardovia omnicolens*, and *Veillonella montpellierensis*.
Allison R. Perrotta 2020	suspected or confirmed by imaging or previous surgery	endometriosis	do not have endometriosis or any inflammatory conditions	Two months before surgery and during the menstrual period and follicular phase	Microbiota composition predicts the stage of endometriosis, and *anaerococcus* has the most positive correlation with the stage of disease. The distribution of vaginal community state types differs between the follicular and menses phases of the menstrual cycle.
Rasheed M. Salah 2013	physician-diagnosed	Endometriosis (with infertility)	asymptomatic fertile women	/	The prevalence of BV was very high in infertile women, particularly those with PCOS and unexplained infertility, not endometriosis.
Bryan A. Wee 2017	physician-diagnosed	Endometriosis (with infertility needing ART or fertility)	without endometriosis	At the time of hysteroscopy	A trend for two organisms to be more abundant or prevalent in the endometriosis Cases (*Ureaplasma spp*., P = 0.042 (vagina) and *G*. *vaginalis*, P = 0.044 (cervix); unadjusted). Four of the five women with a history of infertility (Cases) colonized with *Ureaplasma* also had a vaginal CST III dominated by *L*. *iners* (P = 0.015). CSTs dominated by *Lactobacillus* were present in the majority of specimens.
Weixia Wei 2020	surgery and pathology	endometriosis	gynecological benign tumor	3–7 days after the menstrual period (early follicular phase)	The lower reproductive tract (CL, CU, and CV) was dominated by *Lactobacillus* belonging to type II. In ET and PF, higher diversity was presented, and types IV and V occurred more frequently. A significant difference in community diversity began showing in the CV of endometriosis patients and gradually increased upward in the reproductive tract. It indicates the microbiota in cervical samples is expected to predict the risk of endometriosis.
Janet D. Wilson 2002	unclear	Endometriosis (with infertility needing IVF)	asymptomatic fertile women	immediately before egg collection	A significantly raised rate of BV in women with tubal factor infertility supports the association between BV, PID, and tubal damage. This cross-sectional study does not help distinguish whether the increased BV rate is secondary to previous tubal damage initiated by *C*. *trachomatis* or *N*. *gonorrhea*, or whether BVAB compromises host immunity and facilitates the spread of these STDs into the upper genital tract, or whether BV itself can cause damage to the fallopian tube.

Note: **AM**: Adenomyosis; **CPP**: Chronic pelvic pain; **ART**: Assisted reproductive technology; **IVF**: In vitro fertilization; **PCR**: Polymerase chain reaction; **NGS**: Next-generation sequencing; **PCOS**: **Polycystic** ovary syndrome; **CL**: Lower third of vagina; **CU**: Posterior fornix; **CV**: Cervical mucus; **ET**: Endometrium; **PF**: Peritoneal fluid; **PID**: Pelvic inflammatory disease; **STD**: Sexually transmitted disease.

The quality of the eight studies incorporated into our review was evaluated using the aforementioned Newcastle-Ottawa Scale (NOS) and the Agency for Healthcare Research and Quality (AHRQ) checklist. The scores assigned to these studies varied, ranging from 4 to 8, with an average score of 6.5. This indicates a moderate overall quality of the selected research. The detailed quality assessment scores, which consider factors such as selection, comparability, exposure, and outcome for cohort and case-control studies, as well as the thoroughness and clarity of reporting in cross-sectional studies, are methodically presented in Tables 3 and 4. These tables provide a breakdown of individual study scores, offering a transparent view of the strengths and limitations inherent in the body of research evaluated.

**Table 3:** NOS for cohort and case-control study.

Study	Study type	Selection	Comparability	Exposure /Outcome	Score
Baris Ata 2019	cohort	***	*	**	7
Rasheed M. Salah 2013	cohort	***	*	**	6
Kanoko Akiyama 2019	case-control	***	**	***	8
Bryan A. Wee 2017	case-control	***	*	**	6
Weixia Wei 2020	case-control	***	*	***	7

Note: The NOS uses a semi-quantitative star rating system that assigns a maximum of nine stars to each of the eight detailed criteria in these three categories, with each

“*” representing one point.

**Table 4:** AHRQ checklist for cross-sectional study.

study item^$^	Xiaopei Chao 2021	Allison R. Perrotta 2020	Janet D. Wilson 2002
1	Yes	Yes	Yes
2	Yes	Yes	Yes
3	Yes	Yes	Yes
4	Unclear	Unclear	No
5	No	No	Unclear
6	No	No	No
7	Yes	Yes	Unclear
8	Yes	Yes	Unclear
9	Yes	Yes	No
10	Yes	Yes	Yes
11	No	No	No
**Score:**	7	7	4

Note: $ represents the 11 items on the AHRQ checklist. A score of 1 is assigned for "YES" and 0 is for "NO" or "Unclear" in response to the items.

### Results of statistical analysis

#### Association between dysbiosis of vaginal microecology and endometriosis

**Fig 2:**
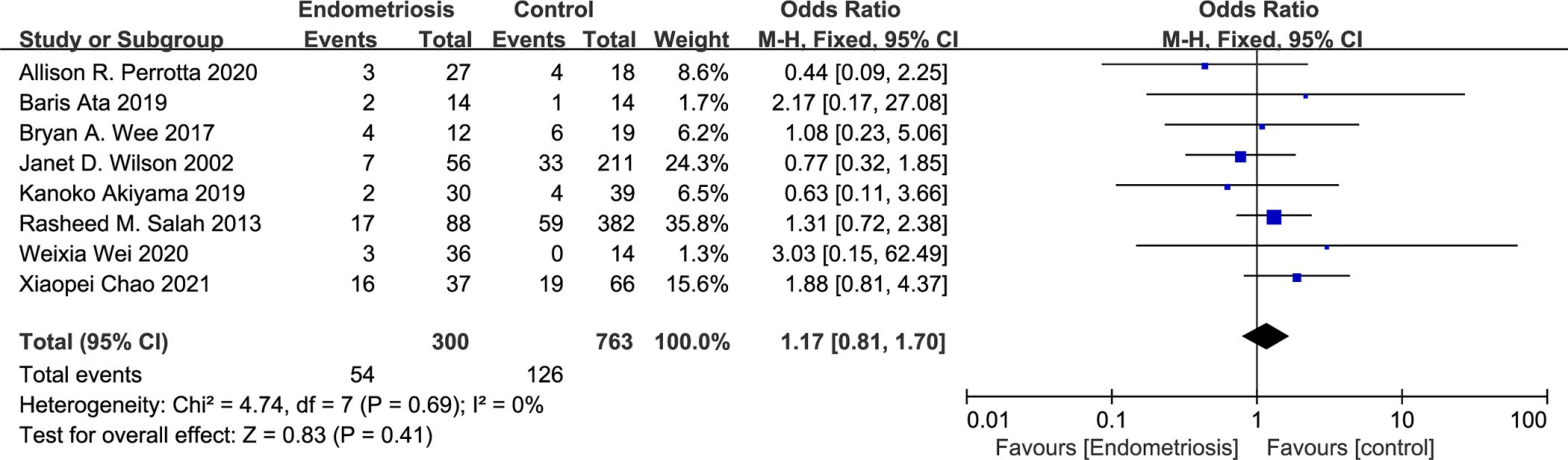
Forest plot of correlation between dysbiosis and endometriosis.

The relationship between vaginal microecology dysbiosis and endometriosis was quantitatively assessed across eight studies. In light of the low heterogeneity observed among these studies, a fixed-effects model was applied to the meta-analysis. The synthesized data yielded a pooled Odds Ratio (OR) of 1.17, with a 95% Confidence Interval (CI) ranging from 0.81 to 1.70, and an I^2^ value indicative of non-existent heterogeneity (I^2^ = 0%) (Fig 2). This OR suggests a positive association between dysbiosis and endometriosis. Nonetheless, the association did not reach statistical significance, prompting considerations of potential clinical and biological implications, as well as the need for further research to clarify this relationship.

#### Detailed analysis of dysbiosis categories and endometriosis

**Fig 3:**
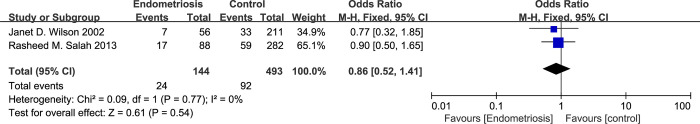
Forest plot of correlation between BV and endometriosis.

In the nuanced analysis of dysbiosis, when the condition was sub-categorized into Bacterial Vaginosis (BV) and Intermediate BV, distinct associations with endometriosis were observed. For BV, the meta-analysis revealed an Odds Ratio (OR) of 0.86 with a 95% Confidence Interval (CI) from 0.52 to 1.41, and an I^2^ of 0%, indicating no observed heterogeneity (Fig 3). This result points to a non-significant inverse association with endometriosis, although this finding is based on a limited number of studies.

**Fig 4:**
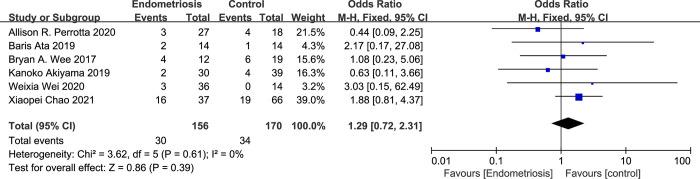
Forest plot of correlation between intermediate BV and endometriosis.

Conversely, for Intermediate BV, the calculated OR was 1.29 (95%CI 0.72–2.31; I^2^ = 0%) (Fig 4), suggesting a positive correlation with endometriosis. However, similar to the findings for BV, the result was not statistically significant, and given the data were derived from a small subset of studies, this association should be interpreted with caution.

#### Association between normal vaginal microecology and endometriosis

**Fig 5:**
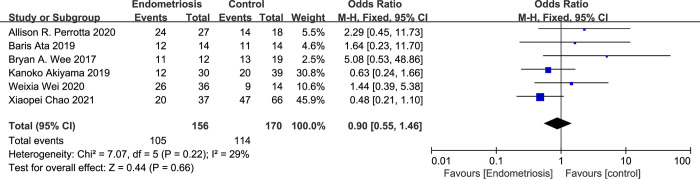
Forest plot of correlation between normal vaginal microecology and endometriosis.

Within our meta-analysis, six studies provided data on the presence of normal vaginal microecology and its potential link to endometriosis. The aggregation of this data, through the application of a fixed-effects model, yielded a pooled Odds Ratio (OR) of 0.90 with a 95% Confidence Interval (CI) spanning from 0.55 to 1.46. The I^2^ value for this analysis was 29% (Fig 5), suggesting a low to moderate level of heterogeneity among the included studies.

The OR indicates a marginally inverse relationship between normal vaginal microecology and the incidence of endometriosis, although this association did not reach statistical significance. Although the evidence is insufficient, this finding suggests that normal vaginal flora may have a protective effect against endometriosis.

#### Subgroup analysis

Subgroup analyses were meticulously conducted to explore the association between dysbiosis of vaginal microecology and endometriosis across various study designs, geographic locations, control group characteristics, and diagnostic methods. The results of these analyses are encapsulated in Table 5.

**Table 5:** Summary of subgroup analyses.

Subgroup	No. of studies	Pooled OR	95% CI	P value
**Study design**				0.95
cohort	2	1.13	0.49–2.57	
case-control	3	0.89	0.21–3.71	
cross-sectional	3	1.00	0.62–1.61	
**Subjects from**				0.39
Asians	5	1.18	0.70–2.01	
non-Asians	3	0.83	0.46–1.53	
**Control group**				0.81
asymptomatic fertile women	6	1.03	0.68–1.55	
gynecological benign tumor	2	0.82	0.14–4.83	
**Diagnose method**				0.90
16S rRNA gene sequencing	6	1.04	0.59–1.83	
modified Spiegel’s criteria	2	0.99	0.57–1.74	
**Dysbiosis degree**				0.90
Intermediate BV	6	1.04	0.59–1.83	
BV	2	0.99	0.57–1.74	

Fixed-effects models were utilized for all subgroup analyses. When categorized by study type, the following pooled Odds Ratios (ORs) were observed:

- Cohort studies: OR 1.13 (95%CI 0.49–2.57) from two studies.
- Case-control studies: OR 0.89 (95%CI 0.21–3.71) from three studies.
- Cross-sectional studies: OR 1.00 (95%CI 0.62–1.61) from three studies.

Further subgrouping by region showed:

- Asian studies (five studies): OR 1.18 (95%CI 0.70–2.01).
- Non-Asian studies (three studies): OR 0.83 (95%CI 0.46–1.53).

When considering the control group composition, the ORs were:

- Healthy fertile women: OR 1.03 (95%CI 0.68–1.55).
- Women with benign gynecologic neoplasms: OR 0.82 (95%CI 0.14–4.83).

Diagnostic methods also formed subgroups, with:

- 16S rRNA gene sequencing: OR 1.04 (95%CI 0.59–1.83).
- Modified Spiegel’s criteria: OR 0.99 (95%CI 0.57–1.74).

The ORs for dysbiosis categorized into intermediate BV and BV were consistent with the results from the diagnostic method subgroups, aligning with the non-significant trends observed in the broader analysis.

Notably, the results of the cohort studies and the Asian subgroup suggest a positive association between dysbiosis and endometriosis. However, it is imperative to emphasize that none of the subgroup analyses reached statistical significance, indicating that while trends can be observed, they do not provide conclusive evidence of a relationship.

### Sensitivity analysis to evaluate stability

A sensitivity analysis was conducted to test the robustness of our findings. Initially, the data were re-analyzed using a random-effects model to account for any potential variability across studies. This analysis yielded an Odds Ratio (OR) of 1.18 (95%CI 0.81–1.72) for dysbiosis and an OR of 0.99 (95%CI 0.52–1.88) for normal vaginal microecology, both consistent with the primary analysis and indicating stability in the results.

Further sensitivity testing involved the sequential exclusion of each study from the meta-analysis. For dysbiosis, the pooled OR values fluctuated minimally, ranging from 1.04 (95%CI 0.68–1.59) to 1.30 (95%CI 0.86–1.98). Similarly, for normal vaginal microecology, the ORs ranged from 0.79 (95%CI 0.47–1.32) to 1.25 (95%CI 0.68–2.31). These narrow ranges confirm that the overall conclusions of our meta-analysis remain unaffected by any single study, indicating a high level of stability in the results.

The detailed findings of the sensitivity analysis, including the impact of each study on the overall effect size, are systematically presented in Table 6.

**Table 6:** Results of sensitivity analysis.

Exclude study	Dysbiosis		Normal	
	OR	95%CI	OR	95%CI
None	1.17	0.81–1.70	0.90	0.55–1.46
Kanoko Akiyama 2019	1.21	0.83–1.07	1.01	0.57–1.79
Baris Ata 2019	1.15	0.79–1.69	0.86	0.52–1.43
Xiaopei Chao 2021	1.04	0.68–1.59	1.25	0.68–2.31
Allison R. Perrotta 2020	1.24	0.85–1.82	0.81	0.49–1.36
Rasheed M. Salah 2013	1.09	0.68–1.76	/	/
Bryan A. Wee 2017	1.18	0.80–1.73	0.79	0.47–1.32
Weixia Wei 2020	1.15	0.79–1.68	0.83	0.49–1.41
Janet D. Wilson 2002	1.30	0.86–1.98	/	/

### Assessment of publication bias

**Fig 6:**
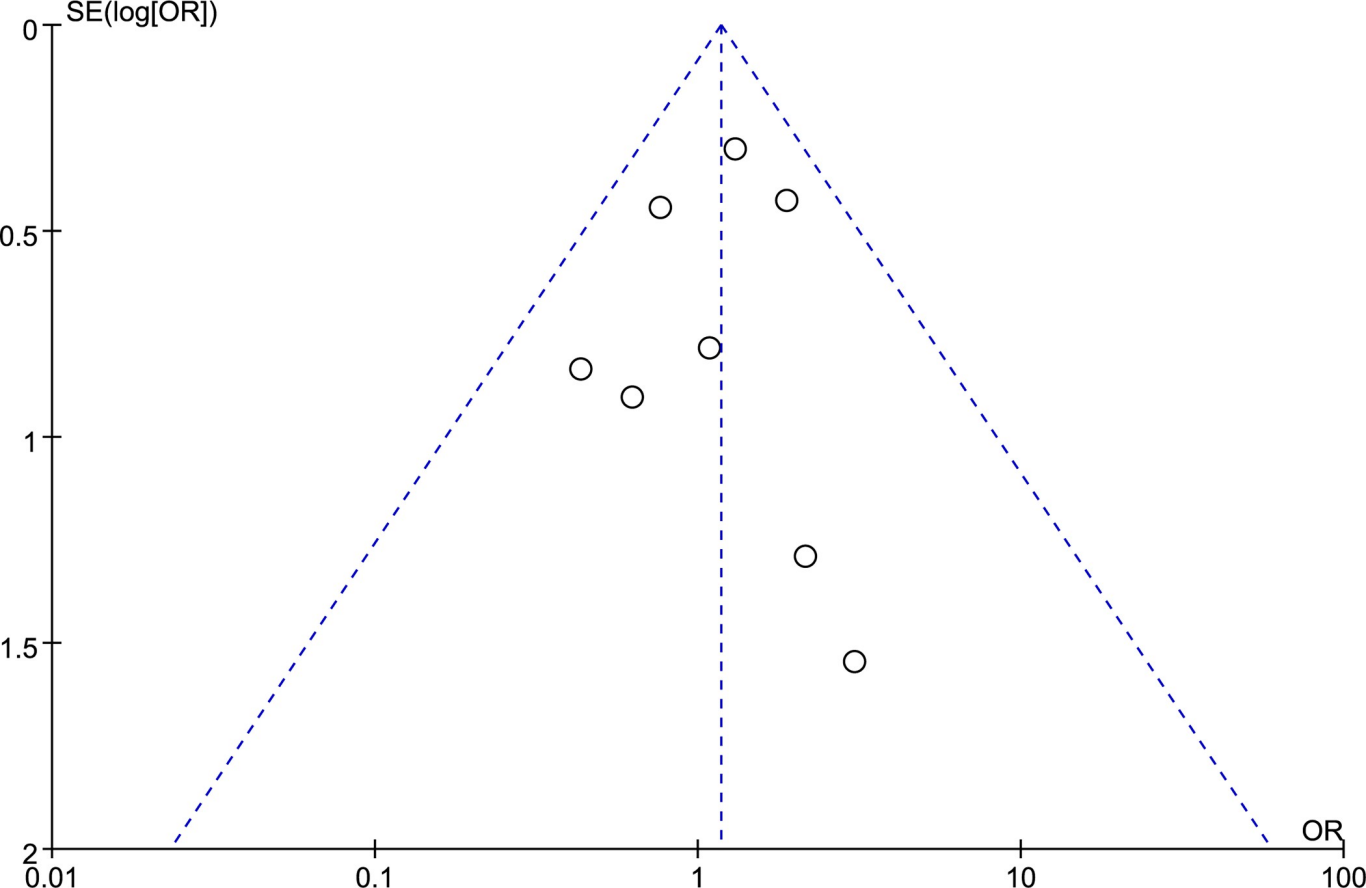
Funnel plots for dysbiosis with endometriosis.

To assess the presence of publication bias within our meta-analysis, we conducted a visual inspection using a funnel plot. The symmetric distribution of the included studies around the combined effect size in the funnel plot suggests the absence of significant publication bias (Fig 6). This graphical tool is instrumental in identifying bias by plotting the effect sizes against a measure of study precision, typically the standard error. The anticipated funnel shape, where studies are evenly distributed around the mean effect size, was observed, implying that the meta-analysis results are likely to be free of bias.

The absence of publication bias reinforces the validity of our findings, indicating that the likelihood of non-publication of small or unfavorable studies is low. This adds to the robustness of our conclusions and suggests that the pooled estimates of association are representative of the available evidence.

## Discussion

This review adds quantitative analysis to the existing literature suggesting a link between the absence of *Lactobacillus*, the proliferation of Bacterial Vaginosis-Associated Bacteria (BVAB) in the cervical-vaginal microbiota, and associations with endometriosis and infertility [41]. To our knowledge, this is the inaugural study to systematically quantify the correlation between dysbiosis of vaginal microecology and endometriosis, uncovering evidence of a positive correlation, particularly with intermediate BV.

The symbiotic evolution of humans with their microbiota over roughly 500 million years has fostered diverse ecological niches, including the oral cavity, gut, skin, and the female genital tract. As our comprehension of microorganisms has advanced from morphological to molecular understanding, pivotal initiatives such as the Human Microbiome Project and the Integrative Human Microbiome Project have laid the groundwork for current microbiome research [42–44]. Despite the significance of the vaginal microbiota, which comprises about 9% of the total human microbiota and has profound implications for reproduction and public health, it has traditionally received less attention compared to oral or gut flora [45].

Bacterial Vaginosis is the most common lower genital tract infection among women of reproductive age. Its clinical presentations often include abnormal discharge, a distinct odor, and discomfort, with a notable proportion of asymptomatic cases, paralleling the asymptomatic nature of some endometriosis cases [45]. BV’s microbiology is complex, typified by a reduction in *Lactobacillus* and an increase in anaerobic bacteria. This shift correlates strongly with various subgroups of BVAB [41]. Similar to BV, endometriosis is not dominated by a single pathogen.

Historically considered sterile, the upper genital tract has, through more recent research into endometriosis, been shown to harbor bacterial colonization, including *Lactobacillus*, *Gardnerella vaginalis*, *Streptococcus*, and *Prevotella bivia* [46–48]. Yet, most genital tract microbiota studies in women with endometriosis have concentrated on the cervix and vagina [36, 49–52]. A review posits that endometriosis pathogenesis may involve an initial infection followed by sterile inflammation, with heightened inflammatory cytokines and innate immunity markers such as Lipopolysaccharides and Toll-like receptor 4 indicating a link between bacterial infection and endometriotic proliferation [21]. The “bacterial contamination hypothesis” also suggests the involvement of *E*. *coli* in menstrual blood and microbial colonization in the endometrium as factors in endometriosis growth [53].

The high heterogeneity of endometriosis poses challenges in its diagnosis, which is typically confirmed through surgical exploration, and its treatment, often leading to a high recurrence rate even with combined surgical and medical intervention. This underscores the need for early or non-invasive diagnostic methods and innovative treatment modalities. Some studies have explored the use of the vaginal microbiome to predict endometriosis stages, especially advanced stages, hinting at an intrinsic connection [39, 54].

As bacterial or inflammatory factors are implicated in endometriosis, novel treatment approaches such as antibiotics, *Lactobacillus* supplementation, and even vaginal microbial transplantation are being explored. For instance, antibiotic treatment has shown efficacy in preventing and reducing endometriosis lesions in animal models [5]. A randomized, double-blind, placebo-controlled study demonstrated the potential of *L*. *gasseri* OLL2809 in preventing endometriosis tissue growth [55]. Despite the differences in vaginal flora between humans and animals, these findings offer promise and warrant further investigation [56]. While oral probiotics have not been shown to alter the vaginal microbiome composition [57], clinical trials indicate that Lactobacillus can mitigate pain and enhance the quality of life for endometriosis patients to some extent [58, 59].

Our meta-analysis corroborates the association between vaginal microecology dysbiosis and endometriosis. Although Next-Generation Sequencing is an advanced technology for studying vaginal flora, its application has been limited. Two included studies employed the Spiegel criteria for BV diagnosis. Some research suggests that vaginal microbiota stability is not solely defined by taxonomic shifts, thus dynamic observation of the vaginal flora in endometriosis patients may be warranted. The Nugent score remains a widely accepted standard for BV diagnosis; however, it can be influenced by the examiner’s subjective assessment, highlighting the need for the application of advanced molecular techniques in future studies. The patients with endometriosis in the eight included studies were not identical, ranging from those with endometriosis alone to those with endometriosis combined with infertility or both, and most of the included studies were silent on the severity of the disease, with one study including III-IV endometriosis and a pilot study for predicting r-ASRM staging. However, whether the vaginal microbiota influences the onset or progression of endometriosis cannot be definitively explained, especially since the vaginal microbiota is constantly changing with time, dysbiosis, or degree of inflammation.

There are limitations in our study, including variability in the types of original studies, racial and ethnic differences, control groups, diagnostic methods, and degrees of vaginal microecology dysbiosis. These factors could impact the comparability of the study groups. Furthermore, the ethnicity of the patient population, primarily of Asian descent in our study, can significantly influence microbiota testing results.

## Conclusion

This meta-analysis provides insights into the potential relationship between dysbiosis of vaginal microecology and endometriosis, indicating a non-significant positive correlation that is particularly notable in cases of intermediate Bacterial Vaginosis (BV). While our findings shed light on this possible connection, the current body of evidence is limited by a variety of factors, including methodological diversity among studies, heterogeneity of patient populations, and differing diagnostic criteria.

The subtle yet consistent trends observed across the reviewed studies highlight the need for a more nuanced understanding of the endogenous interactions between vaginal microecology and endometriosis. To confirm and clarify the nature of these associations, further research employing high-quality, standardized methodologies is essential. Future studies should aim to elucidate the underlying biological mechanisms, with a focus on longitudinal designs that can better address questions of causality and the potential for therapeutic interventions targeting vaginal microecology.

In summary, the hypothesis that alterations in vaginal microecology may play a role in the pathogenesis or progression of endometriosis is compelling but not yet definitively supported by the available evidence. Future investigations must build on this preliminary understanding to fully unravel the complexities of this association.

## Supporting information

S1 Checklist PRISMA 2020 checklist. (DOCX)

S1 File Search strategy. (DOCX)

S2 File Specific quality assessments. (DOCX)

S3 File The results of subgroup analyses. (ZIP)

S1 Table Detailed basic characteristics. (XLSX)
