# Supplementary material for: Correlation between dysbiosis of vaginal microecology and endometriosis: A systematic review and meta-analysis
Source: PLoS One. 2024 Jul 8;19(7):e0306780. doi: 10.1371/journal.pone.0306780 (PMC11230536; doi:10.1371/journal.pone.0306780)
Supplement: S1 Checklist — (DOCX) [file pone.0306780.s001.docx]

| **Section and Topic** | **Item #** | **Checklist item** | **Location where item is reported** |
| --- | --- | --- | --- |
| **TITLE** | | |  |
| Title | 1 | The report is identified as a systematic review and meta-analysis | Page 1 |
| **ABSTRACT** | | |  |
| Abstract | 2 | The abstract includes Background (including objective), Methods, Results, Conclusions, and Registration number. | Page 2-3 |
| **INTRODUCTION** | | |  |
| Rationale | 3 | Described in the Introduction. | Page 4-7 |
| Objectives | 4 | Described in the Introduction. | Page 7 |
| **METHODS** | | |  |
| Eligibility criteria | 5 | Described in the Methods. | Page 9 |
| Information sources | 6 | Described in the Methods. | Page 8 |
| Search strategy | 7 | Described in the Methods. | Page 7-8 |
| Selection process | 8 | Described in the Methods. | Page 8-10 |
| Data collection process | 9 | Described in the Methods. | Page 12 |
| Data items | 10a | Described in the Methods. | Page 12 |
|  | 10b | Described in the Methods. | Page 12 |
| Study risk of bias assessment | 11 | Described in the Methods. | Page 11-12 |
| Effect measures | 12 | Described in the Methods. | Page 12 |
| Synthesis methods | 13a | Described in the Methods. | Page 12-13 |
|  | 13b | Described in the Methods. | Page 12-13 |
|  | 13c | Described in the Methods. | Page 12-13 |
|  | 13d | Described in the Methods. | Page 12-13 |
|  | 13e | Described in the Methods. | Page 12-13 |
|  | 13f | Described in the Methods. | Page 12-13 |
| Reporting bias assessment | 14 | Described in the Methods. | Page 13 |
| Certainty assessment | 15 | Described in the Methods. | Page 13 |
| **RESULTS** | | |  |
| Study selection | 16a | Described in the Results. | Page 13-14 |
|  | 16b | Described in the Results. | Page 13-14 |
| Study characteristics | 17 | Described in the Results. | Page 14-16 |
| Risk of bias in studies | 18 | Described in the Results. | Page 18-19 |
| Results of individual studies | 19 | Described in the Results. | Page 16-17 |
| Results of syntheses | 20a | Described in the Results. | Page 19-20 |
|  | 20b | Described in the Results. | Page 20-21 |
|  | 20c | Described in the Results. | Page 21-22 |
|  | 20d | Described in the Results. | Page 22-23 |
| Reporting biases | 21 | Described in the Results. | Page 24-25 |
| Certainty of evidence | 22 | Described in the Results. | Page 23-24 |
| **DISCUSSION** | | |  |
| Discussion | 23a | Described in the Discussion. | Page 25-29 |
|  | 23b | Described in the Discussion. | Page 25-29 |
|  | 23c | Described in the Discussion. | Page 25-29 |
|  | 23d | Described in the Discussion. | Page 29 |
| **OTHER INFORMATION** | | |  |
| Registration and protocol | 24a | Registration number: CRD42023448280. | Page 3, 7 |
|  | 24b | [PROSPERO (york.ac.uk)](https://www.crd.york.ac.uk/PROSPERO/) | Page 7 |
|  | 24c | The protocol is described in the Methods. | Page 7-13 |
| Support | 25 | Non-financial support. | N |
| Competing interests | 26 | The authors declare that they have no competing interests | N |
| Availability of data, code and other materials | 27 | All data generated or analyzed during this study are included in this published article and its supplementary information files. | N |

*From:*  Page MJ, McKenzie JE, Bossuyt PM, Boutron I, Hoffmann TC, Mulrow CD, et al. The PRISMA 2020 statement: an updated guideline for reporting systematic reviews. BMJ 2021;372:n71. doi: 10.1136/bmj.n71

For more information, visit: <http://www.prisma-statement.org/>
