# Supplementary material for: Correlation between dysbiosis of vaginal microecology and endometriosis: A systematic review and meta-analysis
Source: PLoS One. 2024 Jul 8;19(7):e0306780. doi: 10.1371/journal.pone.0306780 (PMC11230536; doi:10.1371/journal.pone.0306780)
Supplement: S1 File — (DOCX) [file pone.0306780.s002.docx]

**Supplementary files 1 Search strategy**

Database: N=1072 Time: 2023.8.15

PubMed：n=117

Search: **(((Lower genital tract) OR (vagina)) AND (((((((("Dysbiosis"[Mesh]) OR ((((((((((Dysbioses) OR (Disbiosis)) OR (Disbioses)) OR (Dys-symbiosis)) OR (Dys symbiosis)) OR (Dys-symbioses)) OR (Dysbacteriosis)) OR (Dysbacterioses)) OR (Disbacteriosis)) OR (Disbacterioses))) OR (("Inflammation"[Mesh]) OR ((((Inflammations) OR (Innate Inflammatory Response)) OR (Inflammatory Response, Innate)) OR (Innate Inflammatory Responses)))) OR (("Infections"[Mesh]) OR (((((Infection and Infestation) OR (Infestation and Infection)) OR (Infections and Infestations)) OR (Infestations and Infections)) OR (Infection)))) OR (("Vaginosis, Bacterial"[Mesh]) OR (((((((((Bacterial Vaginitides) OR (Vaginitides, Bacterial)) OR (Bacterial Vaginosis)) OR (Vaginitis, Nonspecific)) OR (Nonspecific Vaginitis)) OR (Bacterial Vaginoses)) OR (Vaginoses, Bacterial)) OR (Bacterial Vaginitis)) OR (Vaginitis, Bacterial)))) OR (Aerobic vaginitis)) OR (("Candidiasis, Vulvovaginal"[Mesh]) OR ((((((((((((((((Vulvovaginal Candidiasis) OR (Moniliasis, Vulvovaginal)) OR (Vulvovaginal Moniliasis)) OR (Genital Vulvovaginal Candidiasis)) OR (Candidiasis, Genital Vulvovaginal)) OR (Vulvovaginal Candidiasis, Genital)) OR (Vaginitis, Monilial)) OR (Monilial Vaginitis)) OR (Candidiasis, Genital)) OR (Genital Candidiasis)) OR (Vaginal Yeast Infections)) OR (Infection, Vaginal Yeast)) OR (Infections, Vaginal Yeast)) OR (Yeast Infection, Vaginal)) OR (Yeast Infections, Vaginal)) OR (Vaginal Yeast Infection)))) OR (("Trichomonas Vaginitis"[Mesh]) OR (((((((Trichomonas Vaginitides) OR (Vaginitides, Trichomonas)) OR (Vaginitis, Trichomonas)) OR (Trichomoniasis, Human)) OR (Human Trichomoniases)) OR (Human Trichomoniasis)) OR (Trichomoniases, Human))))) AND (("Endometriosis"[Mesh]) OR (((Endometrioses) OR (Endometrioma)) OR (Endometriomas)))**

**Embase：n=860**

**#19**#3 AND #4 AND #18

**#18**#5 OR #6 OR #7 OR #8 OR #9 OR #10 OR #11 OR #12 OR #13 OR #14 OR #15 OR #16 OR #17

**#17'trichomonal fluor'**/exp OR **'trichomonal fluor'** OR **'trichomonas vaginalis vaginitis'**/exp OR **'trichomonas vaginalis vaginitis'** OR **'trichomonas vaginitis'**/exp OR **'trichomonas vaginitis'** OR **'trichomoniasis vaginalis'**/exp OR **'trichomoniasis vaginalis'** OR **'vaginal trichomoniasis'**/exp OR **'vaginal trichomoniasis'**

**#16'vaginal trichomoniasis'**/exp

**#15'candida vulvovaginitis'**/exp OR **'candida vulvovaginitis'** OR **'candidal vulvovaginitis'**/exp OR **'candidal vulvovaginitis'** OR **'candidiasis, vulvovaginal'**/exp OR **'candidiasis, vulvovaginal'** OR **'monilial vulvovaginitis'**/exp OR **'monilial vulvovaginitis'** OR **'vulvo-vaginal candidiasis'**/exp OR **'vulvo-vaginal candidiasis'** OR **'vulvo-vaginal candidosis'**/exp OR **'vulvo-vaginal candidosis'** OR **'vulvovaginal candidosis'**/exp OR **'vulvovaginal candidosis'** OR **'vulvovaginal moniliasis'**/exp OR **'vulvovaginal moniliasis'** OR **'vulvovaginitis caused by candida'**/exp OR **'vulvovaginitis caused by candida'** OR **'vulvovaginitis due to candida'**/exp OR **'vulvovaginitis due to candida'** OR **'vulvovaginal candidiasis'**/exp OR **'vulvovaginal candidiasis'**

**#14'vulvovaginal candidiasis'**/exp

**#13'aerobic vaginitis'**/exp

**#12'acute vaginitis'**/exp OR **'acute vaginitis'** OR **'bacterial vaginitis'**/exp OR **'bacterial vaginitis'** OR **'bacterial vaginosis'**/exp OR **'bacterial vaginosis'** OR **'colpitis'**/exp OR **'colpitis'** OR **'kolpitis'**/exp OR **'kolpitis'** OR **'nonspecific vaginitis'**/exp OR **'nonspecific vaginitis'** OR **'vagina infection'**/exp OR **'vagina infection'** OR **'vagina inflammation'**/exp OR **'vagina inflammation'** OR **'vaginal infection'**/exp OR **'vaginal infection'** OR **'vaginitis, acute'**/exp OR **'vaginitis, acute'** OR **'vaginosis, bacterial'**/exp OR **'vaginosis, bacterial'** OR **'vaginitis'**/exp OR **'vaginitis'**

**#11'vaginitis'**/exp

**#10'accidental infection'**/exp OR **'accidental infection'** OR **'acute infection'**/exp OR **'acute infection'** OR **'autoinfection'**/exp OR **'autoinfection'** OR **'bacterial infections and mycoses'**/exp OR **'bacterial infections and mycoses'** OR **'bacteroid infection'**/exp OR **'bacteroid infection'** OR **'chain of infection'**/exp OR **'chain of infection'** OR **'focal infection'**/exp OR **'focal infection'** OR **'infection mechanism'**/exp OR **'infection mechanism'** OR **'infection route'**/exp OR **'infection route'** OR **'infection, focal'**/exp OR **'infection, focal'** OR **'infections'**/exp OR **'infections'** OR **'infectious disease'**/exp OR **'infectious disease'** OR **'infectivity'**/exp OR **'infectivity'** OR **'route of infection'**/exp OR **'route of infection'** OR **'infection'**/exp OR **'infection'**

**#9'infection'**/exp

**#8'acute inflammation'**/exp OR **'acute inflammation'** OR **'inflammation reaction'**/exp OR **'inflammation reaction'** OR **'inflammation response'**/exp OR **'inflammation response'** OR **'inflammatory condition'**/exp OR **'inflammatory condition'** OR **'inflammatory lesion'**/exp OR **'inflammatory lesion'** OR **'inflammatory process'**/exp OR **'inflammatory process'** OR **'inflammatory reaction'**/exp OR **'inflammatory reaction'** OR **'inflammatory response'**/exp OR **'inflammatory response'** OR **'inflammatory syndrome'**/exp OR **'inflammatory syndrome'** OR **'reaction, inflammation'**/exp OR **'reaction, inflammation'** OR **'response, inflammatory'**/exp OR **'response, inflammatory'** OR **'inflammation'**/exp OR **'inflammation'**

**#7'inflammation'**/exp

**#6'dysbacteriosis'**/exp OR **'dysbacteriosis'** OR **'dysbiosis'**/exp OR **'dysbiosis'**

**#5'dysbiosis'**/exp

**#4'lower genital tract'** OR **'vagina'**/exp OR **'vagina'**

**#3**#1 OR #2

**#2'adenomyosis externa'**/exp OR **'adenomyosis externa'** OR **'endometriosis externa'**/exp OR **'endometriosis externa'** OR **'endometriosis'**/exp OR **'endometriosis'**

**#1'endometriosis'**/exp

**SCOPUS**: n=7

Refined: All open access

Search:( TITLE-ABS-KEY ( **symbiosis** OR inflammation OR infections OR ''bacterial AND vaginitides'' OR ''aerobic AND vaginitis'' OR ''vulvovaginal AND candidiasis'' OR ''trichomonas AND vaginitis'' ) AND TITLE-ABS-KEY ( endometriosis ) )

**The Cochrane Library：n=4**


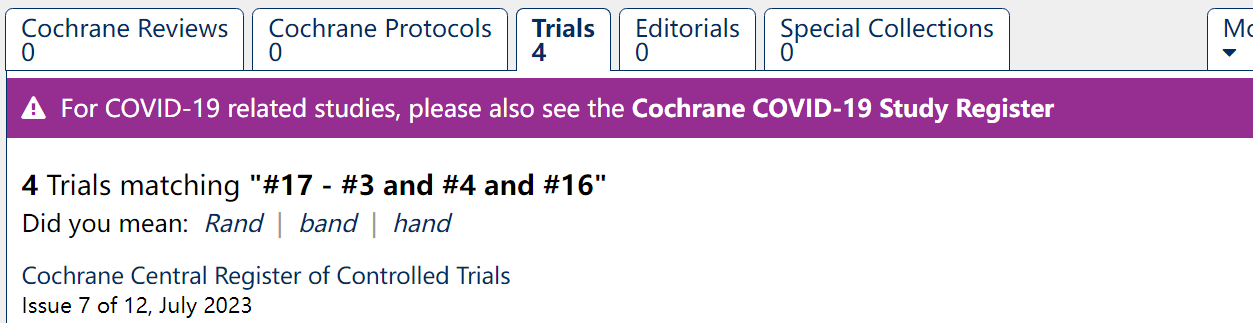


**
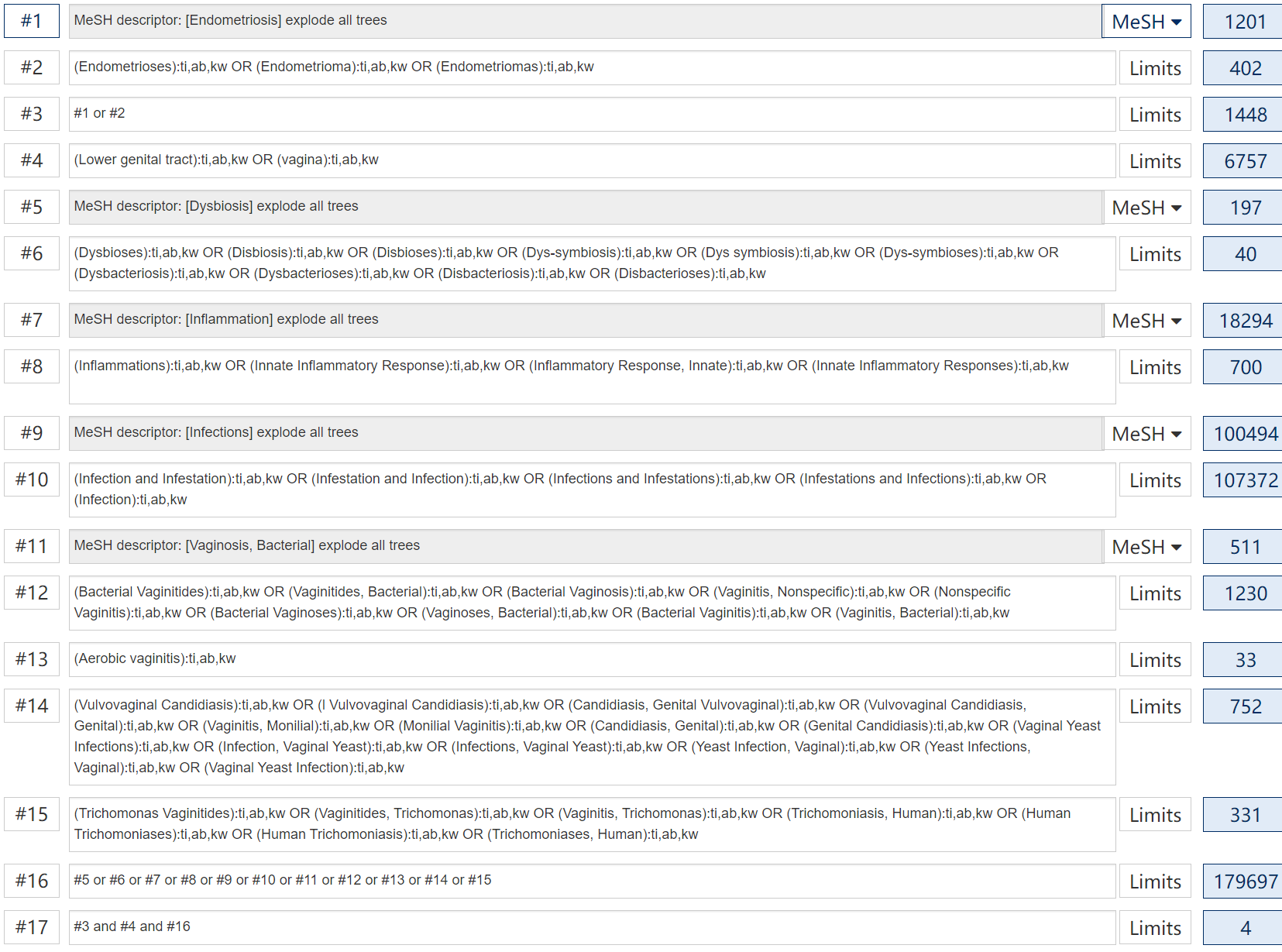
**

**MEDLINE**（ovid）：n=64

|  | **检索内容** | **结果** | **检索方式** |
| --- | --- | --- | --- |
| 1 | (Endometriosis or Endometrioses or Endometrioma or Endometriomas).af. | 34119 | 高级 |
| 2 | (Lower genital tract or vagina).af. | 55223 | 高级 |
| 3 | (Dysbiosis or Dysbioses or Disbiosis or Disbioses or Dys-symbiosis or Dys symbiosis or Dys-symbioses or Dysbacteriosis or Dysbacterioses or Disbacteriosis or Disbacterioses).af. | 19390 | 高级 |
| 4 | (Inflammation or Inflammations or Innate Inflammatory Response or Inflammatory Response, Innate or Innate Inflammatory Responses).af. | 738889 | 高级 |
| 5 | (Vaginosis, Bacterial or Bacterial Vaginitides or Vaginitides, Bacterial or Bacterial Vaginosis or Vaginitis, Nonspecific or Nonspecific Vaginitis or Bacterial Vaginoses or Vaginoses, Bacterial or Bacterial Vaginitis or Vaginitis, Bacterial).af. | 5619 | 高级 |
| 6 | Aerobic vaginitis.af. | 170 | 高级 |
| 7 | (Candidiasis, Vulvovaginal or Vulvovaginal Candidiasis or Moniliasis, Vulvovaginal or Vulvovaginal Moniliasis or Genital Vulvovaginal Candidiasis or Candidiasis, Genital Vulvovaginal or Vulvovaginal Candidiasis, Genital or Vaginitis, Monilial or Monilial Vaginitis or Candidiasis, Genital or Genital Candidiasis or Vaginal Yeast Infections or Infection, Vaginal Yeast or Infections, Vaginal Yeast or Yeast Infection, Vaginal or Yeast Infections, Vaginal or Vaginal Yeast Infection).af. | 4480 | 高级 |
| 8 | (Trichomonas Vaginitis or Trichomonas Vaginitides or Vaginitides, Trichomonas or Vaginitis, Trichomonas or Trichomoniasis, Human or Human Trichomoniases or Human Trichomoniasis or Trichomoniases, Human).af. | 3734 | 高级 |
| 9 | (((((((((Infections or Infection) and Infestation) or Infestation) and Infection) or Infections) and Infestations) or Infestations) and Infections) or Infection).af. | 1603763 | 高级 |
| 10 | 3 or 4 or 5 or 6 or 7 or 8 or 9 | 2266978 | 高级 |
| 11 | 1 and 2 and 10 | 64 | 高级 |

**BIOSIS**(Ovid): n=0

|  | **检索内容** | **结果** | **检索方式** |
| --- | --- | --- | --- |
| 1 | (Endometriosis or Endometrioses or Endometrioma or Endometriomas).af. | 5 | 高级 |
| 2 | (Lower genital tract or vagina).af. | 13 | 高级 |
| 3 | (Dysbiosis or Dysbioses or Disbiosis or Disbioses or Dys-symbiosis or Dys symbiosis or Dys-symbioses or Dysbacteriosis or Dysbacterioses or Disbacteriosis or Disbacterioses).af. | 5 | 高级 |
| 4 | (Inflammation or Inflammations or Innate Inflammatory Response or Inflammatory Response, Innate or Innate Inflammatory Responses).af. | 82 | 高级 |
| 5 | (((((((((Infections or Infection) and Infestation) or Infestation) and Infection) or Infections) and Infestations) or Infestations) and Infections) or Infection).af. | 1492 | 高级 |
| 6 | (Vaginosis, Bacterial or Bacterial Vaginitides or Vaginitides, Bacterial or Bacterial Vaginosis or Vaginitis, Nonspecific or Nonspecific Vaginitis or Bacterial Vaginoses or Vaginoses, Bacterial or Bacterial Vaginitis or Vaginitis, Bacterial).af. | 1 | 高级 |
| 7 | Aerobic vaginitis.af. | 0 | 高级 |
| 8 | (Candidiasis, Vulvovaginal or Vulvovaginal Candidiasis or Moniliasis, Vulvovaginal or Vulvovaginal Moniliasis or Genital Vulvovaginal Candidiasis or Candidiasis, Genital Vulvovaginal or Vulvovaginal Candidiasis, Genital or Vaginitis, Monilial or Monilial Vaginitis or Candidiasis, Genital or Genital Candidiasis or Vaginal Yeast Infections or Infection, Vaginal Yeast or Infections, Vaginal Yeast or Yeast Infection, Vaginal or Yeast Infections, Vaginal or Vaginal Yeast Infection).af. | 0 | 高级 |
| 9 | (Trichomonas Vaginitis or Trichomonas Vaginitides or Vaginitides, Trichomonas or Vaginitis, Trichomonas or Trichomoniasis, Human or Human Trichomoniases or Human Trichomoniasis or Trichomoniases, Human).af. | 0 | 高级 |
| 10 | 3 or 4 or 5 or 6 or 7 or 8 or 9 | 1572 | 高级 |
| 11 | 1 and 2 and 10 | 0 | 高级 |

Wanfang： n=20

文献类型：全部

限制条件：英文

**检索表达式（中英文扩展&主题词扩展）：** 主题:(子宫内膜异位症 OR 子宫内膜异位囊肿 OR 巧克力囊肿 OR EMs OR DIE) and 主题:(下生殖道 OR 阴道) and 主题:(微生态失调 OR 微生态失衡 OR 菌群失常 OR 炎症 OR 感染 OR 细菌性阴道病 OR 需氧菌性阴道炎 OR 念珠菌性阴道炎 OR 霉菌性阴道炎 OR 滴虫性阴道炎)

CNKI：n=0
