# Supplementary material for: Correlation between dysbiosis of vaginal microecology and endometriosis: A systematic review and meta-analysis
Source: PLoS One. 2024 Jul 8;19(7):e0306780. doi: 10.1371/journal.pone.0306780 (PMC11230536; doi:10.1371/journal.pone.0306780)
Supplement: S2 File — (DOCX) [file pone.0306780.s003.docx]

**Cohort Study: NOS Scale**

|  | 条目 | 评价标准 | Baris Ata | Rasheed M. Salah |
| --- | --- | --- | --- | --- |
| 研究人群选择 | 暴露组的代表性如何 | 1. 真正代表人群中暴露组的特征；* 2. 一定程度上代表了人群中暴露组的特征；* 3. 选择某类人群，如护士、志愿者； 4. 未描述暴露组来源情况。 | * | * |
|  | 非暴露组的选择方法 | 1. 与暴露组来自同一人群；* 2. 与暴露组来自不同人群； 3. 未描述非暴露组来源情况。 |  |  |
|  | 暴露因素的确定方法 | 1. 固定的档案记录（如外科手术记录）；* 2. 采用结构式访谈；* 3. 研究对象自己写的报告； 4. 未描述。 | * | * |
|  | 确定研究起始时尚无要观察的结局指标 | 1. 是；* 2. 否。 | * | * |
| 组间可比性 | 设计和统计分析时考虑暴露组和未暴露组的可比性 | 1. 研究控制了最重要的混杂因素；* 2. 研究控制了任何其他的混杂因素（此条可修改用以说明特定控制第二重要因素）。* | * | * |
| 结果测量 | 结果测量 | 1.盲法独立评价；* 2. 有档案记录；* 3. 自我报告； 4. 未描述。 | * | * |
|  | 结果发生后随访是否足够长 | 1. 是（评价前规定恰当的随访时间）；* 2. 否。 |  |  |
|  | 暴露和非暴露组的随访是否充分 | 1. 随访完整；* 2. 有少量研究对象失访但不至于引入偏倚（规定失访率或描述失访情况）；* 3. 有失访（规定失访率）但未行描述； 4. 未描述随访情况。 | * | * |

**Case-control Study: NOS Scale**

|  | 条目 | 评价标准 | Kanoko Akiyama | Bryan A. Wee | Weixia Wei |
| --- | --- | --- | --- | --- | --- |
| 研究人群选择 | 研究人群选择 | 1. 恰当，有独立的确定方法和人员；* 2. 恰当，如基于档案记录或自我报告； 3. 未描述。 | * | * | * |
|  | 病例的代表性 | 1. 连续或有代表性的系列病例；* 2. 有潜在的选择偏倚或未描述。 | * | * | * |
|  | 对照的选择 | 1. 与病例同一人群的对照；* 2. 与病例同一人群的住院人员为对照； 3. 未描述。 |  |  |  |
|  | 对照的确定 | 1. 无目标疾病史（端点）；* 2. 未描述来源。 | * | * | * |
| 组间可比性 | 设计和统计分析时考虑病例和对照的可比性 | 1. 研究控制了最重要的混杂因素；* 2. 研究控制了任何其他的混杂因素（此条可以进行修改用以说明特定控制第二重要因素）* | ** | * | * |
| 暴露因素的测 量 | 暴露因素的确定 | 1. 固定的档案记录（如外科手术记录）；* 2. 采用结构式访谈且不知访谈者是病例或对照； 3. 采用未实施盲法的访谈（即知道病例或对照的情况）；未描述 | * | * | * |
|  | 采用相同的方法确定病例和对照组暴露因素 | 1. 是；* 2. 否。 | * | * | * |
|  | 无应答率 | 1. 病例和对照组无应答率相同；* 2. 描述了无应答者的情况； 3. 病例和对照组无应答率不同且未描述。 | * |  | * |

**Cross-sectional Study: AHRQ Checklist**

"Yes" is 1 point, "No" or "Unclear" are 0 points, out of a total of 11 points. With 0-3 being low quality, 4-7 medium quality, and 8-11 high quality.

**Xiaopei Chao 2021：7**

|  | 条目 | Yes | No | Unclear |
| --- | --- | --- | --- | --- |
| 1 | 是否明确了资料的来源（调查、文献回顾）？ | * |  |  |
| 2 | 是否列出了暴露组和非暴露组（病例和对照）的纳入及排除标准或参考以往的出版物？ | * |  |  |
| 3 | 是否给出了鉴别患者的时间阶段？ | * |  |  |
| 4 | 如果不是人群来源的话，研究对象是否连续？ |  |  | * |
| 5 | 评价者的主观因素是否掩盖了研究对象其他方面情况？ |  | * |  |
| 6 | 描述了任何为保证质量而进行的评估（如对主观结局指标的检测/再检测） |  | * |  |
| 7 | 解释了排除分析任何患者的理由 | * |  |  |
| 8 | 描述了如何评价和（或）控制混杂因素的措施 | * |  |  |
| 9 | 如果可能，解释了分析中是如何处理丢失数据的 | * |  |  |
| 10 | 总结了患者的应答率及数据收集的完整性 | * |  |  |
| 11 | 如果有随访，查明预期患者不完整数据所占的百分比或随访结果 |  | * |  |

|  |
| --- |

**Allison R. Perrotta 2020：7**

|  | 条目 | Yes | No | Unclear |
| --- | --- | --- | --- | --- |
| 1 | 是否明确了资料的来源（调查、文献回顾）？ | * |  |  |
| 2 | 是否列出了暴露组和非暴露组（病例和对照）的纳入及排除标准或参考以往的出版物？ | * |  |  |
| 3 | 是否给出了鉴别患者的时间阶段？ | * |  |  |
| 4 | 如果不是人群来源的话，研究对象是否连续？ |  |  | * |
| 5 | 评价者的主观因素是否掩盖了研究对象其他方面情况？ |  | * |  |
| 6 | 描述了任何为保证质量而进行的评估（如对主观结局指标的检测/再检测） |  | * |  |
| 7 | 解释了排除分析任何患者的理由 | * |  |  |
| 8 | 描述了如何评价和（或）控制混杂因素的措施 | * |  |  |
| 9 | 如果可能，解释了分析中是如何处理丢失数据的 | * |  |  |
| 10 | 总结了患者的应答率及数据收集的完整性 | * |  |  |
| 11 | 如果有随访，查明预期患者不完整数据所占的百分比或随访结果 |  | * |  |

| **Janet D. Wilson 2002：4** |
| --- |

|  | 条目 | Yes | No | Unclear |
| --- | --- | --- | --- | --- |
| 1 | 是否明确了资料的来源（调查、文献回顾）？ | * |  |  |
| 2 | 是否列出了暴露组和非暴露组（病例和对照）的纳入及排除标准或参考以往的出版物？ | * |  |  |
| 3 | 是否给出了鉴别患者的时间阶段？ | * |  |  |
| 4 | 如果不是人群来源的话，研究对象是否连续？ |  | * |  |
| 5 | 评价者的主观因素是否掩盖了研究对象其他方面情况？ |  |  | * |
| 6 | 描述了任何为保证质量而进行的评估（如对主观结局指标的检测/再检测） |  | * |  |
| 7 | 解释了排除分析任何患者的理由 |  |  | * |
| 8 | 描述了如何评价和（或）控制混杂因素的措施 |  |  | * |
| 9 | 如果可能，解释了分析中是如何处理丢失数据的 |  | * |  |
| 10 | 总结了患者的应答率及数据收集的完整性 | * |  |  |
| 11 | 如果有随访，查明预期患者不完整数据所占的百分比或随访结果 |  | * |  |
